# Understanding Antibody Responses in Early Life: Baby Steps towards Developing an Effective Influenza Vaccine

**DOI:** 10.3390/v13071392

**Published:** 2021-07-17

**Authors:** Elene A. Clemens, Martha A. Alexander-Miller

**Affiliations:** Department of Microbiology and Immunology, Wake Forest School of Medicine, Winston-Salem, NC 27101, USA; eclemens@wakehealth.edu

**Keywords:** newborn, influenza virus, vaccine, B cell, Tfh, antibody

## Abstract

The immune system of young infants is both quantitatively and qualitatively distinct from that of adults, with diminished responsiveness leaving these individuals vulnerable to infection. Because of this, young infants suffer increased morbidity and mortality from respiratory pathogens such as influenza viruses. The impaired generation of robust and persistent antibody responses in these individuals makes overcoming this increased vulnerability through vaccination challenging. Because of this, an effective vaccine against influenza viruses in infants under 6 months is not available. Furthermore, vaccination against influenza viruses is challenging even in adults due to the high antigenic variability across viral strains, allowing immune evasion even after induction of robust immune responses. This has led to substantial interest in understanding how specific antibody responses are formed to variable and conserved components of influenza viruses, as immune responses tend to strongly favor recognition of variable epitopes. Elicitation of broadly protective antibody in young infants, therefore, requires that both the unique characteristics of young infant immunity as well as the antibody immunodominance present among epitopes be effectively addressed. Here, we review our current understanding of the antibody response in newborns and young infants and discuss recent developments in vaccination strategies that can modulate both magnitude and epitope specificity of IAV-specific antibody.

## 1. Influenza Virus Infection of Young Infants

Following birth, infants must make the transition from the protected environment of the womb to the antigen-rich outside world. This requires substantial adjustment by the immune system as it begins to encounter its first non-self antigens. Shortly after birth, the immune system exists in an altered state that is broadly characterized by the suppression of inflammatory responses, which recent studies suggest is necessary for the induction of tolerance to commensal microbiota and harmless environmental antigens [1,2,3]. While this benefits the long-term health of the individual, these immune alterations can leave young infants vulnerable to viral and bacterial illnesses [4,5]. Influenza A virus (IAV) is a relatively common viral respiratory tract pathogen that exhibits higher rates of infection and leads to higher incidence of serious disease or secondary complications following primary infection of newborns and young infants [6,7,8].

Compounding this increased susceptibility to infection and severe disease, the dampened newborn immune response poses significant challenges for the elicitation of protective immunity by vaccination [9]. In addition to lacking potency in the initial immune response, responses in young infants tend to be relatively transient, limiting the window of protection that is conferred even in the face of successful immune stimulation [10,11]. Because of these barriers to vaccine efficacy, there is currently no IAV vaccine available to infants under 6 months of age [12,13]. As vaccination is currently the most effective method of preventing IAV infection and ameliorating severe disease, this leaves infants without the benefit of vaccine-mediated protection outside of passively transferred maternal antibody, which wanes significantly within the first 2–3 months after birth, with minimal remaining antibody by 7 months [14,15,16,17]. Given the requirement for two doses of the IAV to achieve protection and the inability to deliver the first dose until 6 months of age, there is a strong and urgent need to better understand not only the nature of viral immunity in newborns and young infants, but how it can be manipulated to provide optimal protection at early times following birth. 

While the immune system of the young infant provides its own special challenges with regard to vaccine development, current approaches towards vaccinating against influenza viruses are far from perfect—even for healthy adults. Influenza viruses are highly variable due to their capacity for antigenic drift, necessitating yearly updates to maintain vaccine efficacy. This requires accurate prediction of circulating strains each season prior to vaccine production [18,19,20]. Current seasonal vaccines contain either inactivated influenza viruses or live attenuated viruses. The former is administered intramuscularly and the latter intranasally. Both types of vaccines are quadrivalent, containing the two strains of influenza A and two strains of influenza B predicted to be most highly circulating that season. Antibodies generated following vaccination are predominantly directed to the variable head region of the influenza hemagglutinin (HA) protein [21,22].

The challenge of annual reformulation of the vaccine has led to considerable effort dedicated to the development of a “universal flu vaccine”—one that elicits immune protection against a wide variety of viral strains. Generation of broadly protective immune responses is of particular interest for young infants considering the potentially life-long consequences of immune responses during this immunologically impressionable period in early life. Opportunely, many of the strategies being investigated in attempts to elicit broadly cross-protective antibodies to influenza viruses may also have a role in improving immune responses in young infants. Here, we will review some of the prominent deficits in the antibody responses of young infants (summarized in Table 1) and discuss recent advances and current considerations in the study of young infant IAV vaccination. We discuss findings in animal models, primarily mice, as well as humans, predominantly cord blood. We note that while the mouse is a highly tractable and powerful model, there are limitations. Mice are born with immune systems that are less mature than humans, not reflecting humans until approximately a week of age. Their rapid aging to adulthood limits the study of immune system maturation through the period of infancy, i.e., newborns versus young infants. Further, differences in maternal antibody transfer, the balance of leukocyte subsets, Toll-like receptors, and antibody subsets may also impact findings in newborn mouse models [23,24]. While these differences must be considered, the mouse provides an important model, especially for in vivo studies. 

We will primarily discuss alterations identified in circulating and secondary lymphoid tissues given our focus on vaccines and that most of our understanding is derived from these sites. However, we note that mucosal immune responses are a critical component of protection following infection and understanding the regulation of immune responses in these tissues in newborns and young infants is a significant area that merits further study.

## 2. The antibody Response of Young Infants

### 2.1. Natural Antibody against Influenza Virus

The B cell compartment of young infants exhibits significant alterations in its innate as well as adaptive compartments. In mouse models, “natural” antibodies are produced by B1 cells, a more innate-like subset of B cells [25,26,27]. There is controversy around the existence of B1 cells in humans and if present, whether their function parallels that reported in mouse models. Additional studies will be required to resolve this issue. 

Natural IgM (nIgM) produced by B1 cells tends to be polyreactive, which may have benefit for protection against a highly variable virus like IAV; however, this is balanced by the limited capacity of B1 cells to undergo affinity maturation to produce high-affinity antibody and to undergo class-switch recombination (CSR) [25]. It appears that a wave of natural antibody producing B1 cells emerge early in life, while B2 cells experience a delay, potentially providing essential protective coverage during the first days of life [28,29,30,31]. 

In mouse models, B1 cells are proposed to recognize conformational epitopes on carbohydrate-rich IAV spike proteins, likely HA [32] and these antibodies often have hemagglutinin (HA)-inhibiting properties [33]. Data showing the ability of natural antibody to promote recruitment and activation of the innate immune system as well as its capacity to inhibit viral entry make it an important potential contributor to the early immune response [32]. In the context of influenza virus infection in mice, the presence of pre-existing virus-reactive IgM has been shown to delay morbidity and mortality via complement-mediated viral neutralization [26,34,35,36,37]. However, these B1-derived antibodies do not increase following influenza virus infection and therefore these cells may have limited utility as targets of vaccination [36]. 

### 2.2. B2 Activation in Response to Influenza Virus

The generation of high-titer, antigen-specific antibody following infection or vaccination is the result of the activation and differentiation of B2 cells. In young infants, deficits are evident at multiple steps in this process. With that noted, there is some debate regarding the relative contributions of inherent B cell dysfunction versus the influence exerted by accessory immune cells to the altered nature of the neonatal B cell response [12,38,39,40,41]. B cells from newborns and young infants display several unique qualities not seen in mature, immunocompetent humoral responses. Beyond global deficiencies in antibody production, one qualitative differences in newborn and adult responses is the change in BCR repertoire and immunoglobulin chain usage in very early life—while this may not affect the quantity of antibody produced, it may certainly influence how different antigens are recognized and responded to [38,40]. 

Due to a lack of antigen exposure, most newborn B cells are naïve cells that express IgM [42]. B cells from cord blood have been shown to have higher surface expression of IgM than those found in adult PBMC, while adults had higher levels of IgM stored intracellularly [43]. In studies of human cord blood cells or newborn mice, ligation of the BCR is capable of eliciting robust proliferation, although there may be deficiencies in downstream activation events, such as surface expression of MHC-II and survival after rapid cell cycle entry [44,45,46]. This is consistent with a model in which newborn B cells can respond rapidly to antigen encounters, but lack the reserve to sustain robust immune responses. Thus, while this transient response may provide some protection against pathogens, it is less conducive to the generation of immune memory or sustained antibody. 

Although neonatal B cells appear capable of responding to antigen via BCR signaling, they have altered responses to both pathogen-associated molecular patterns (PAMPs) and cytokines produced by other immune cells, often exerting anti-inflammatory effects in response to what would normally be activating signals [47,48]. For example, while B cells from human newborns have been shown to have similar expression of TLRs as those from adult peripheral blood [49], in both mouse and humans, they display distinct patterns of cytokine production after stimulation with TLR agonists [48,49]. In addition to limiting inflammation, this altered cytokine profile also likely contributes to the Th2 bias characteristic of newborn immunity [49,50]. 

In terms of direct alterations in B cell effector function, defects in the BAFF/APRIL signaling system lead to reduced differentiation into plasma cells in mice, thereby limiting the actual production of antibody [47]. Human cord blood B cells also have diminished expression of CD25, with correspondingly low sensitivity to IL-2 stimulation, resulting in limited proliferative responses [44]. Thus, these cells exhibit a defective response to host-produced immune factors as well as pathogen-derived innate stimuli. 

B cell effector function is also dependent on the ability to mobilize towards sites where antigen and accessory cells are present. Cord blood B cells exhibit reduced expression of both CD62L and CCR7, leading to a reduced capacity of newborn B cells to home to lymphoid organs and obtain T cell help [44]. In contrast, no difference between cord blood and adult B cell expression of CXCR5, which is required for movement both to the follicle and to the germinal center (GC), was observed. The reduced activation and localization of B cells to lymphoid tissue both dampen the initial antibody response and severely limit the potential to elicit lasting, high-quality protective antibody and B cell memory requiring affinity maturation. 

### 2.3. The Germinal Center of Young Infants

As noted above, the B cell repertoire of young infants is more limited than adults. This is the result of low expression of the enzyme TdT, which leads to decreased non-templated-nucleotide addition during V(D)J recombination [51,52]. Thus, young infants have a restricted array of BCR from which to initiate a response. The generation of effective lasting immunity requires optimization of pathogen recognition by selection of high-affinity antibody and the generation of memory B cells (MBCs) as well as long-lived plasma cells (LLPCs). These processes are driven by differentiation and affinity maturation in the GC. 

The intricacies of clonal selection—particularly as it pertains to selection of epitope specificity rather than simply BCR avidity—are still not fully understood. The GC reaction requires both spatial and temporal coordination between several subsets of immune cells and as a result there are multiple facets of this response that can be altered in young infants. The establishment, expansion, and function of GCs is diminished in this age group, leading to decreased quantity and quality of protective immune responses after infection or vaccination [10,53,54,55]. 

Some of the defects in affinity maturation can be attributed to intrinsic alterations in B cell function. B cells from human infants exhibit a reduction in somatic hypermutation (SHM) [56,57]. SHM allows for generation of high affinity BCR that promotes clonal selection as a results of successful competition for T cell help in the GC reaction. In a study of neutralizing antibodies generated in young human infants (<3 months of age) following infection with respiratory syncytial virus (RSV), investigators found limited evidence of SHM [58].

B cells isolated from cord blood also have demonstrated defects in the purine salvage pathway, resulting in an increased reliance upon de novo nucleotide synthesis [59]. This metabolic strain is speculated to restrict processes with high nucleotide requirements, such as proliferation and SHM [59]. Once GC B cells have mutated their receptors to obtain antigen, they must also test their ability to obtain survival signals from T follicular helper (Tfh) cells; however, B cells from murine newborns and young infants have a decreased capacity to present antigen to receive T cell help, further limiting affinity maturation of the overall response by reducing opportunities for positive selection [60].

In addition to intrinsic alterations in B cells, the GC response in young infants is limited by a variety of external factors integral to GC function. GC development requires structural organization by a network of follicular dendritic cells (FDCs) and follicular reticular cells (FRC), which are not fully mature during the early postnatal period [61,62]. The hampered ability to organize the anatomic architecture required for a GC reaction appears to not only delay the onset, but also limit persistence and diminish the magnitude of affinity matured antibody responses in newborn mice [63]. FDC immaturity is also associated with diminished availability of captured and displayed immune complexes in the GC, further limiting the capacity of B cells to take up and present antigen to acquire T cell help. 

As mentioned above, Tfh cells play an essential role in affinity maturation. This T cell subset is characterized by expression of CXCR5, which recognizes CXCL13 secreted by GC FDCs to allow homing to the germinal center, as well as the key transcription factors Bcl-6, Ascl2, and Batf [64]. Tfh are responsible for positive selection of B cell clones that can efficiently take up, process, and present antigen in the light zone of the GC. Signaling through ICOS/ICOSL and CD40/CD40L inhibit B cell apoptosis during T/B interactions facilitated by TCR recognition of presented antigen [65]. These signals also drive the differentiation of GC B cells into memory B cells (MBCs) and long-lived plasma cells (LLPCs); however, the mechanisms of this process are incompletely understood [66]. Recent studies in humans have described a subset of circulating Tfh (cTfh) in the periphery that correlate with the affinity and magnitude of antibody responses to IAV vaccination [67,68,69,70,71]. These cells serve as a surrogate for assessment of GC Tfh cells. The importance of Tfh cells both in and out of the germinal center has made their study an area of considerable interest in the field of vaccinology. 

The ability of Tfh to provide help to B cells in the GC depends on coordination of Tfh differentiation, positioning, and expression of appropriate surface receptors. Tfh in young infants, however, exhibit functional immaturity that is suspected to be a major limiting factor in the newborn antibody response. The distinct transcriptional profile of newborn Tfh is most notably associated with decreased expression of the master regulator Bcl-6 and the GC homing cytokine CXCR5, leading to impairments in Tfh development and localization [72,73,74]. Differentiation of Tfh from CD4^+^ T cells is inhibited by factors extrinsic as well as intrinsic to newborn T cells, in part due to the reliance of Tfh on DCs and activated B cells for successful differentiation [73,75]. Both CD4^+^ T cells from newborn mice adoptively transferred into adults as well as adult CD4^+^ T cells adoptively transferred into newborn mice experience defects in Tfh differentiation, expansion and maintenance of GC [73]. Even when they are able to successfully enter the follicle, studies using a mouse model showed newborn-derived follicular T cells tend towards a higher ratio of T follicular regulatory (Tfr):Tfh cells, suggesting more frequent differentiation into a regulatory phenotype as well as skewing towards Th2 and Th17 polarization, which further inhibits the development of robust antibody responses [72,76]. 

One notable characteristics of newborn Tfh and other T cell populations is the decreased surface expression of CD40L [77,78,79], which is integral to the provision of T cell help to B cells. This has been shown in both mice and humans. Interestingly, by 3 weeks of age, human infants gain the capacity to express CD40L following stimulation [79]. There are conflicting reports on whether newborn B cells have correspondingly low levels of CD40 expression, or whether low CD40L expression on Tfh is the limiting factor in the CD40-CD40L interaction [80,81,82]. An assessment of cord blood from both term and preterm births found that the expression of CD40 by B cells collected from term newborns did not differ significantly from that seen in adult B cells [83]. However, CD40 levels were much lower in cord blood collected from preterm births, suggesting that impairment of CD40 expression by B cells may fall on a continuum depending on gestational age [83]. It is also possible that even if newborn B cells have adult-like expression of CD40, they are still be subject to alterations in signal transduction independent of any Tfh-dependent factors.

### 2.4. Long-Lived Plasma Cells

Deficiencies in Tfh function are particularly deleterious to the selection and differentiation of LLPCs, which colonize the bone marrow and constitutively secrete affinity-matured antibody for years to decades after the primary antigen encounter. The provision of T cell help to induce plasma cell differentiation appears to rely on extended cell-to-cell contact between Tfh and B cells in the GC, which is in part dependent on a robust CD40/CD40L interaction and effective B cell presentation of antigen [84]. Murine newborn B cells have been reported to exhibit decreased expression of BAFF/APRIL receptor TACI, which impedes upregulation of CD138 and adoption of a plasma cell phenotype [47]. Considering the alterations in B and Tfh function mentioned above, it is unsurprising that newborns and young infants display dampened differentiation of GC-derived plasma cells compared to their adult counterparts [12,85]. 

The challenges continue for plasma cells that successfully undergo terminal differentiation. Upon egress from the GC, future LLPCs must make their way to the bone marrow (BM) and establish themselves in a BM niche. In addition to a reduced ability of plasma cells from newborns to migrate to the BM, studies in mice suggest that the microenvironment of the BM is less supportive of LLPC colonization and persistence [85]. This inhospitable environment is associated with diminished expression of adhesion molecules and secretion of pro-survival cytokines such as BAFF and APRIL by BM stromal cells, independent from alterations impacting B cell differentiation [11,86,87]. Together, the limited differentiation of plasma cells from the GC and impaired survival of LLPCs in the bone marrow are suspected to be major contributors to the relative transience of circulating antibody following infection or vaccination of newborns and young infants.

### 2.5. Memory B Cells

As newborns have minimal encounters with antigen prior to birth, MBC responses in early life are practically non-existent, as evidenced by the scarcity of CD27^hi^ MBCs in infancy [88]. While this absence of preexisting memory limits the rapid generation of affinity matured antibody following antigen exposure in young infants, early antigen encounters appear to prime for more effective subsequent responses by generating MBCs [12,89,90]. The differentiation of MBCs requires less T cell help, lower receptor affinity, and has lower metabolic demand than is required for differentiation of plasma cells [53]. Recent studies demonstrate that in some cases, memory cells can even form in the absence of germinal centers [91,92,93]. Because of this, the development of MBCs may be less sensitive to the impairments in B cells and the accessory cells present in newborns and young infants compared to LLPCs. 

The more efficient differentiation of MBCs versus plasma cells in young infants does not, however, result in unaltered generation of MBCs. MBCs elicited in early life may have a shorter lifespans than those produced by a mature immune system, as evidenced by the apparent loss of immune memory to hepatitis B in a significant number of adolescents who had received hepatitis B vaccines as newborns [94]. Further exploration of newborn MBC dynamics is warranted to better understand the development and persistence of this key B cell subset. Fortunately, while investigation of MBCs has been technically challenging due to the necessity of examining cellular populations compared to relatively the straightforward serologic assessments that serve as proxies to LLPCs, recent advances in high-throughput sequencing and multiplexed analysis are rapidly expanding our ability to comprehensively assess antigen-specific B cell memory responses [95].

## 3. Strategies to Increase the Effectiveness and Breadth of Protection Conferred by Newborn Influenza Vaccines

From the above discussion, it is clear that a broad range of alterations in the development, activation, and effector function of both B cells and accessory cells is present in the immune system of newborns and young infants. Thus, there is likely no one factor responsible for the dampened antibody responses characteristic of young infant immunity. Because of this, a variety of distinct pathways and molecules may be viable targets for improving antibody responses in these individuals. However, this also means that remediation of a single immune component may not result in the desired outcome if other key aspects of the interconnected response are left unaddressed and thus targeting multiple cells types/pathways may be needed.

A common approach to improving the immune response in young infants is to increase the magnitude of the antibody response to vaccination by inducing sufficient immune activation to compensate for diminished responsiveness [96,97,98]. This approach is supported by findings demonstrating that multiple immune subsets can mount robust responses when removed from the newborn immune microenvironment and provided a surplus of activating signals, whether by adoptive transfer or in vitro stimulation [11,61,82]. Although not the norm for responses in young infants, the capacity for robust responses under select conditions is likely an adaptation to allow a full-fledged protective immune response in the setting of severe or life-threatening infection [99]. In these cases, there is an abundance of accessory immune signals provided by the pathogen in addition to infection-associated damage to tissues that can activate innate immune sensors. 

This highly immunostimulatory environment can be mimicked in the relatively safer setting of vaccination through inclusion of adjuvants. Historically, adjuvants have been considered agents that amplify immune responses by acting non-specifically on innate immune components, thereby indirectly stimulating adaptive immune cells [100]. However, as our understanding evolves, the concept that “more is better” may not always apply given increasing evidence that the newborn immunity is not simply a diminished version of adult immunity, but has fundamental differences in the qualitative responses to certain stimuli. This may be particularly relevant in efforts to develop broadly protective influenza vaccines, where targeting antibody responses to specific epitopes may be of greater interest than general elicitation of high titer influenza virus-specific antibody. 

Highly conserved IAV epitopes, particularly the stem domain of the surface protein HA, have become an appealing target for universal vaccine approaches. However, targeting epitopes that can confer broad protection is challenging even in the setting of a fully mature adult immune system. This is the result of the immune system’s apparent preference to mount immune responses to variable over conserved epitopes. The mechanisms driving the development and enforcement of this immunologic “subdominance” of conserved IAV epitopes are not entirely understood; however, it has been shown to be a dynamic process that is amenable to modulation [101,102,103]. It is speculated that development of epitope specificity in MBC and LLPC populations is likely driven by selection events in the GC, as altering the conditions of the GC reaction influence relative epitope representation. The enforcement of antibody immunodominance, i.e., the focusing of the response on one or a select group of epitopes, appears to rely largely on the ability of B cell clones to obtain the antigen and the T cell help they need for successful affinity maturation, i.e., subdominant clones are less successful in the GC because they are inherently poor competitors [102,104]. 

The factors dictating epitope dominance have not been fully elucidated. There may be a role for epitope accessibility for BCR binding, as dominance does not always correlate to either average BCR avidity or initial precursor frequency [101,105,106]. With that said, the avidity of stem-specific B cells was reported to be lower avidity in a study from Angeletti et al. [102]. In the context of vaccination, immunodominance can be mitigated by reducing competition from dominant clones, either by removing these clones from the GC reaction or by increasing access to subdominant epitopes [104,107]. This is consistent with the success of experimental vaccination approaches utilizing a headless stem construct, wherein removing the immunodominant variable head epitopes allowed expansion of stem-specific clones [108,109,110]. 

Reducing competition for Tfh interactions has also been shown to alleviate immunodominance, i.e., an increased frequency of Tfh in the draining lymph node was associated with improved antibody responses to the HA stem [102]. Furthermore, regulatory activity by Tfr may have a role in dictating immunodominance, potentially through enforcement of stringent B cell selection, which is thought to minimize generation of potentially autoreactive specificities [111,112]. In this regard, stem-reactive antibodies have been shown to have increased polyreactivity [113,114,115] and it is interesting that Tfr appear to promote stem-reactive antibodies in mice in the setting of vaccine boosting [116]. However, in another study a lower ratio of Tfr/Tfh was associated with more stem-specific antibody [102]. Thus, more work is needed to understand the role of Tfr in the selection of individual B cell clones. 

Together, the findings establish a model in which the elicitation of desirable antibody to subdominant epitopes may require a higher threshold of immune activation (increased antigen availability, improved frequency/function of GC B cells and Tfh) to allow a broader diversity of B cell clones to successfully undergo affinity maturation. The factors implicated in promoting alleviation of antibody subdominance closely mirror those implicated as defective in the generation and maintenance of GC responses in young infants that leads to impaired production of lasting antibody. As such, it is unsurprising that many of the strategies under investigation for the promotion of subdominant antibody responses have significant overlap with those that have shown promise for vaccination of young infants. 

Vaccination during early life would increase the chance of the vaccine being the first encounter with IAV, providing an opportunity to influence all subsequent antibody responses, a process termed original antigenic sin (OAS) [117,118]. Further investigation will be necessary to examine how the potential induction of OAS during the neonatal period might be influenced by altered newborn immunity and reduced persistence of immune responses that are elicited in early life. It is possible that the poor formation of immune memory during infancy would decrease lasting OAS effects from newborn vaccination, although it seems likely that even limited memory would be able to influence antibody response to IAV encounters later in life. 

### 3.1. Modulation of Dendritic Cells

As the interface between innate and adaptive immunity, DCs have become a prominent target for modulation of immune responses through vaccination. DCs from CB and newborn mice have functional deficits in their capacity to activate other immune cells, displaying lower levels of surface costimulatory molecules, presenting less antigen, and producing reduced quantities of pro-inflammatory cytokines [60,119,120,121,122,123,124,125,126,127]. Of these, particularly notable is the diminished production of the IL-12p35 subunit of IL-12p70, which is thought to be a major contributing factor to the Th2 bias characteristic of newborn immune responses [119,120,128]. Many efforts to improve newborn antibody responses through use of adjuvants have focused on shifting newborn immunity towards a Th1 response via stimulating DC cytokine production rather than directly targeting T cells.

**Figure 1:**
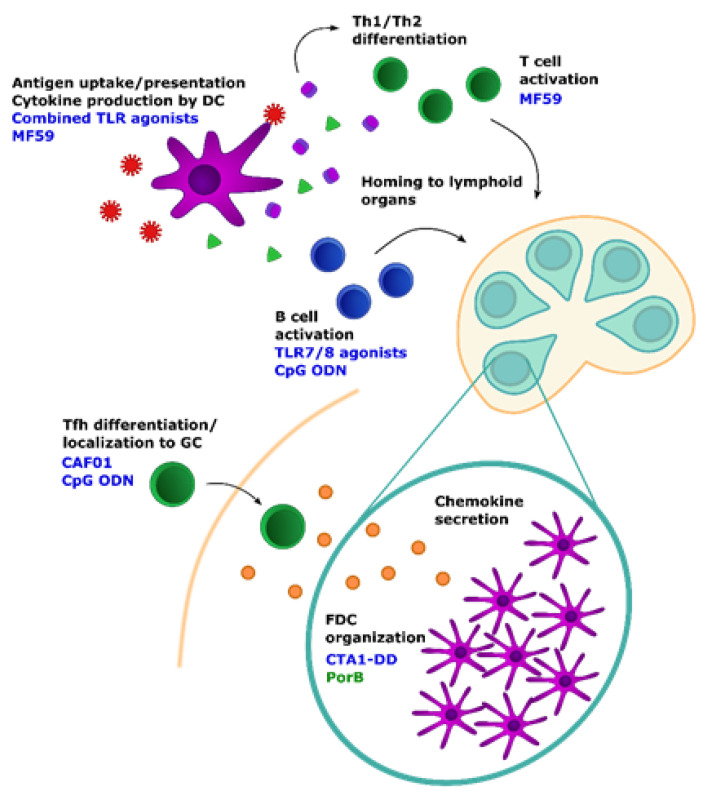
Induction of the germinal center response. Initiation of an affinity matured antibody response requires the coordinated activation and localization of several key immune subsets. Improvements in any of these, including T and B cell activation, differentiation, and homing to lymphoid organs, promote a greater magnitude and diversity of antibody. GC formation in young infants must also overcome defects in the structural framework of the GC that results from FDC immaturity. Increased innate immune stimulation via adjuvants has been shown to confer global benefits for the early steps of the GC formation. Adjuvants in blue have demonstrated effects in newborns or young infants, while those in green have been shown to work in adults. Whether they can work to modulate the indicated responses in young infants is yet to be explored.

The success of the TLR2, 4, and 9-containing Bacille Calmette–Guerin (BCG) vaccine in generating robust Th1 responses and eliciting effective B cell immunity in newborns (for review, see [129]) suggested that TLR agonists may be good adjuvants for targeting DCs (Figure 1). However, in considering this approach, the qualitative alterations in newborn TLR function, despite similar patterns of TLR expression to adult cells [49,130,131,132,133] must be considered. For example, in humans, DC responses to TLR4 and TLR9 are distinct from those seen in adults until at least 6 months of age [134]. In addition, despite a higher ratio of plasmacytoid DCs (pDCs) to conventional DCs (cDCs) than is seen in adults, pDCs have impaired production of type I IFN in response to TLR7 and TLR9 signaling, which is thought to contribute to reduced antiviral responses [122]. 

**Figure 2:**
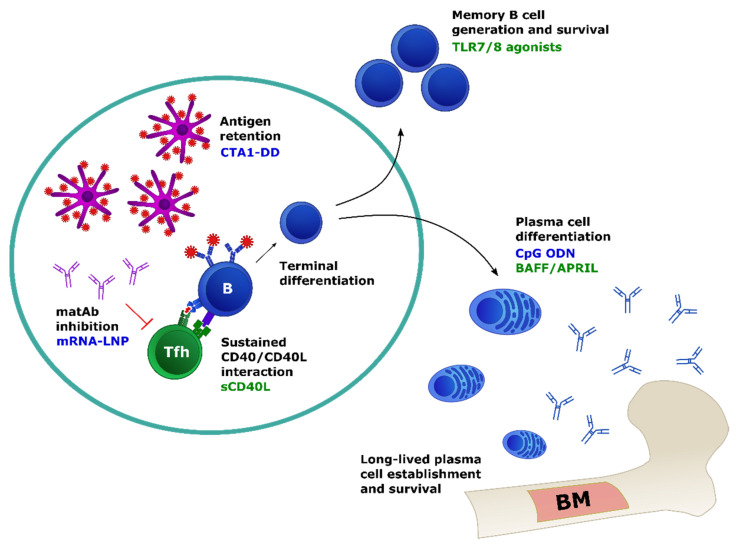
Persistence of GC-derived humoral responses. Prolongation of the germinal center reaction is associated with higher rates of SHM, increased differentiation of LLPCs, and improved generation of broadly neutralizing antibody. Extending the availability of antigen in the GC appears to sustain the GC reaction; if B cells have no antigen to present, they cannot obtain T cell help. Even after successful induction of GC responses, young infants are particularly prone to transient reactions. Once these differentiated B cells exit the germinal center, they face the challenge of continued survival, which is particularly difficult for newborn LLPCs colonizing the immature bone marrow (BM) niche. Adjuvants in blue have demonstrated effects in newborns or young infants, while those in green have been shown to work in adults. Whether they can modulate the indicated responses in young infants is yet to be explored.

One strategy to remedy these deficits has been to combine adjuvants in an attempt to synergize DC activation signals to elicit more adult-like maturation and cytokine production. This may be particularly effective when used to activate different modalities of TLR signaling, i.e., TRIF- versus MyD88-dependent signaling, particularly in terms robust of IL-12 production [123,135,136]. Targeting the endosomal TLRs that sense nucleic acid may also be more effective in circumventing DC deficiencies in young infants [98,137] (Figure 1 and Figure 2). These findings support the efficacy of TLR agonists as effective DC-stimulating adjuvants for young infants; however, it is important that careful consideration be paid to the altered nature of the immune response in choosing these agonists.

Inclusion of the adjuvant MF59 is associated with increased recruitment and maturation of DCs [138] (Figure 1). Antigen-bearing cells have been found in the lymph node as early as 3 hours following administration of antigen and adjuvant [139]. Squalene-based adjuvants have also been shown to enhance antigen uptake by DCs [140]. These data suggest that these adjuvants are effective in generating DCs that can activate T cells.

Use of adjuvants with DC-stimulating activity has also been shown to enhance subdominant antibody responses associated with prime and boost in adult models, including those investigating antibody responses to IAV [141,142,143,144]. It has been speculated that this may be due to increased antigen presentation by DCs, thereby reducing the reliance on MBCs for antigen presentation to T cells during subsequent encounters [145,146]. The association between increased availability of antigen and alleviation of immune subdominance supports a model in which accessibility to factors promoting success in the germinal center can improve the chances that a subdominant clone will be able to expand and differentiate in to either MBCs or LLPCs.

### 3.2. Modulation of Follicular Dendritic Cells (FDCs)

Recent studies have turned attention towards FDCs as an approach to modulate the immune response to vaccines [97,147]. In a recent study, the TLR ligand-based adjuvant PorB was shown to increase presentation of antigen on FDCs in the GC [146] (Figure 1). While it appears that antigen encounter in general can accelerate the maturation of structural components necessary for GC formation [63], Schussek et al. recently demonstrated in a murine neonate model that the adjuvant CTA1-DD can specifically target FDCs through binding to complement receptors [148] (Figure 1). Administration of CTA1-DD conjugated to the M2e protein of influenza virus increased GC generation, T and B cell localization to the GC via CXCL13 signaling, and class-switched antibody production, in addition to reducing morbidity and mortality following lethal IAV infection when conjugated to the M2e protein of influenza virus (Figure 1). Although antibody titers were only followed through 5 weeks, administration of CTA1-DD also seemed to improve the generation of LLPCs. 

Maintenance of the GC relies in part on the sustained provision of antigen by FDCs to B cells, permitting the extensive affinity maturation that appears to be necessary for the generation of LLPCs [149,150,151]. Although this has not been explicitly studied in the context of influenza vaccination, increased availability of antigen and prolonged GC reactions have both been associated with improved antibody responses to subdominant epitopes, including those associated with broadly neutralizing antibodies to HIV [152,153]. Development of broadly reactive memory B cells has also been attributed to persistent germinal center foci that permit extensive V_H_ mutations [154]. It is possible that improved loading of antigen to FDCs would have the combined effects of improving total LLPC generation and alleviating subdominance of desirable conserved IAV epitopes in both MBC and LLPC populations. 

It is possible that young infants do not require extensive SHM to generate cross-reactive antibody to influenza viruses considering that infants and children can generate broadly neutralizing HIV-specific antibody with relatively limited SHM [155,156]. Similarly, several adult-generated antibodies to the HA stem have been found to have minimal SHM, with even a single mutation to the germline sequence conferring high affinity binding [157,158]. It is therefore not unreasonable to speculate that young infants may, in fact, be well equipped to develop cross-strain protection against influenza viruses, particularly given the potentially increased polyreactivity of the newborn BCR repertoire [159]. To this point, we have found strong HA stem-specific responses in newborn NHP following influenza virus infection [160]. 

### 3.3. Modulation of T Follicular Helper Cells

Due to their integral role in the development of high-quality, lasting antibody responses, elicitation of robust Tfh responses using adjuvants has been an area of interest not only for young infants, but for adults as well. In mouse models several adjuvants have been demonstrated to improve Tfh differentiation, frequency, and function in the newborn GC, including CpG oligodeoxynucleotides (ODNs) and the Mincle agonist CAF01 (Figure 1) [73,74,96]. The impact of CpG appears to be through driving Tfh to committed GC Tfh [73,74]. The action of CAF01 has not yet been elucidated. 

In general, efforts to improve Tfh contributions to antibody responses have focused on increasing their frequency and functional capacity. However, there is still much to be learned about the extent to which adjuvants affect Tfh cells directly versus indirectly through modulation of innate immune function. Further investigation is required to determine if our understanding of the relationship between adjuvants and non-follicular CD4 cells is applicable to Tfh cells, as well as how these interactions might be altered in the context of the newborn immune system. For example, stimulation with TLR2 or TLR5 agonists in vitro has been shown to directly stimulate T cells from young infants [161,162,163]; how these agonists may directly impact Tfh differentiation is not known. 

In adult models of infection, increased Tfh frequency—both in the GC and in peripheral blood—is associated with improved antibody responses to IAV vaccination [69,70,71]. As their function is closely tied to clonal selection and affinity maturation in the GC, Tfh have been recently investigated as potential contributors to the development of antibody immunodominance after both infection and vaccination. The frequency of Tfh within the lymph node was directly correlated with antibody responses to the HA stem in a mouse model [102]. Furthermore, MF59, an adjuvant that has been demonstrated to improve early life Tfh responses, has also been shown to improve antibody responses to the HA stem in human adults [141,143]. 

The presence of cross-protective, but subdominant, antibody may also depend on the ratio of Tfh:Tfr in the GC, which may be relevant in the context of the bias towards development of Tregs and Tfr by the newborn immune system [72,116,164]. While it remains unclear whether the relationship between Tfh and immunodominance in young infants will be the same as in adults, the success of Tfh stimulation for elicitation of antibody responses in young infants presents a promising avenue for future development of strategies to generate broadly protective IAV vaccines.

### 3.4. Modulation of B Cells

In addition to the many ways in which adjuvants can indirectly modulate antibody function through their effects on other cells, it is possible for them to act directly on B cells. Similar to Tfh, however, relatively little is known about the direct mechanisms of adjuvant activity on newborn B cells. Although vaccination studies provide crucial information about how different components of the immune system work together to generate practical results, it can be difficult to parse out the precise sites of adjuvant activity. In an attempt to address this in a model using TLR agonist as adjuvants, we have demonstrated that TLR7/8 agonist R848 can directly stimulate the activation of B cells isolated from newborn NHP in vitro [165] (Figure 1). We would expect this finding to carry over to human newborns considering the homology in TLR expression between humans and NHP. In adults, TLR7 signaling in particular is demonstrated to facilitate the generation of MBCs by directly acting on B cells, with subsequently improved generation of secondary plasma cell responses after boost [166,167]. The possible capacity of TLR7/8 signaling to promote B cell memory in young infants is also consistent with our observation that use of R848 as an adjuvant for an inactivated IAV vaccine results in the robust production of HA stem-specific antibody following boost and challenge in the absence of detectable antibody following the initial priming dose [168]. This supports the feasibility of targeting newborn B cells through innate immune signals, even in the context of the altered TLR responsiveness seen in early life [49].

In addition to use of PAMPS that are likely to affect multiple immune populations, small molecules that bind B cell surface receptors involved in survival and proliferation, such as BAFF, APRIL, and soluble CD40L, have shown promise as potential adjuvants in adult models [169] (Figure 2). However, the utility of these adjuvants in young infants may be limited by differences in expression of the surface receptors for these molecules [170]. This is exemplified by the diminished expression of TACI by B cells observed in newborn mice, leading to deficits in plasma cell differentiation even in the presence of exogenous BAFF and APRIL [47]. Importantly, however, this same study showed that TACI could be upregulated by the administration of CpG ODN, suggesting that this adjuvant may promote plasma cell generation (Figure 2). This underscores the importance of developing a nuanced understanding of the immune system of newborns and young infants to promote rational vaccine design that adequately accounts for these unique characteristics.

One important caveat of studies on the efficacy of adjuvant in stimulating newborn antibody responses is the well-established discrepancy between early effector responses and the persistence of protection. Given the earlier time points often assessed in newborn models, it is quite possible that studies examining the induction of antibody responses with vaccine adjuvants may overestimate the long-term protection provided. Additionally, when considering epitope specificity as an important component of the antibody response, it is important to consider the dynamic nature of immunodominance—the antibody profile present shortly after antigen encounter may be very different from that seen after completion of the GC response and establishment of LLPCs in the bone marrow [101]. While limited time courses are a valid practical constraint, the ability to elicit lasting memory and plasma cell responses is an essential component of vaccine efficacy. 

### 3.5. Overcoming Regulation by Maternal Antibody

Vaccination against influenza during pregnancy has undeniable benefits for young infants. However, high titers of passively transferred antibody can be an additional barrier to eliciting antibody responses in young infants. Blunted antibody responses in the presence of maternal antibody have been demonstrated in humans for several routine newborn vaccinations, with the extent of antibody inhibition directly correlating with titers of maternal antibody [17,171]. Unfortunately, the lack of an approved influenza vaccine for infants under 6 months of age has limited our ability to study the effects of maternal antibody on newborn antibody responses in this setting. With that said, the initial studies to determine utility of the trivalent inactivated influenza vaccine (TIV) in infants showed infants between 10–22 weeks of age with maternal antibody did not have significant increases in hemagglutination inhibition assay (HAI) titers following vaccination [172]. It is important to keep in mind, however, that this vaccine was poorly immunogenic in this age group even when maternal antibody was limited and thus the extent of the regulatory effect is difficult to ascertain. Still, evidence of decreased responses to inactivated influenza virus in the presence of maternal antibody has been observed in animal models including infant mice, piglets, and foals [3,173,174,175,176,177]. 

There have been several mechanisms proposed to account for the inhibition of humoral responses in young infants by maternal antibody. In general, it is suspected that limiting antigen availability, through epitope masking and/or antibody-mediated clearance, and inhibition of B cell signaling, termed antibody feedback, results in the faulty activation and priming of antigen-specific B cell clones [171,178]. However, a recent study by Vono et al. using a murine model of IAV vaccination found that while maternal antibody specific for HA inhibited the generation of newborn antibody responses in a dose-dependent manner, the activation of B cells and initial establishment of GCs was unchanged [3]. Instead, the presence of maternal antibody shortened the duration of GC activity, corresponding to deficits in the expansion of Tfh and subsequent generation of MBCs and LLPCs. This proposed model wherein antibody inhibition by maternal antibody is mediated by premature cessation of the GC response is consistent with the findings by Willis et al. that nucleoside-modified mRNA vaccination can alleviate the inhibitory effects of maternal antibody by prolonging the duration of the GC reaction [179] (Figure 2).

Interestingly, maternal antibody appears to shape the specificity of the newborn response. B cells found in the GC when maternally derived antibodies were present used distinct BCRs from those present in the absence of maternal antibody [3]. While BCR usage does not necessarily equate to epitope specificity, it is tempting to speculate that maternal antibody may be shaping epitope recognition of the newborn response. The ability of antibody to a given epitope to inhibit further generation of antibody to that same epitope has been demonstrated both in context of passive antibody transfer and induced antibody responses [101,180]. Further exploration of the differences between maternal and newborn epitope specificity will be particularly relevant to the development of cross-protective influenza vaccines, as the manipulation of maternal immunity may provide another tool that can be used to shape the newborn immune response.

Finally, in contrast the inhibitory effects described above, it is worth noting that there are data supporting the ability of pre-existing antibody to promote responses under some circumstances [181]. In mice, administration of anti-TNP antibody with TNP-BSA or TNP-KLH resulted in antibody responses that were several hundred fold higher than with antigen alone [182,183]. One mechanism by which pre-existing antibody has been reported to promote immune responses is through increased antigen presentation by dendritic cells. This can occur as a result of Fc-mediated uptake [184]. An increase in antigen uptake by DCs has the potential to strongly impact the ability of naïve CD4^+^ T cells to be activated [185], which in turn can support B cell activation, proliferation, survival and isotype switching. Given the complexity of the competing mechanisms, both positive and negative, at play and the differences in antibody across individuals that is transferred to the infant, additional studies are warranted to fully understand the effects of maternal antibody in the context of influenza vaccination of young infants.

## 4. Concluding Remarks

Influenza A virus infections are an annually recurring public health threat due to antigenic variability in the circulating strains and the ability to cause severe pathology, particularly in vulnerable populations such as young infants. As we expand our understanding of immune regulation in these individuals, it has become clear that the challenge of eliciting robust antibody responses in early life is not just in magnifying the responses that are present, but in understanding with precision the functional attributes needed for optimal protection as well as how to overcome the fundamental alterations in how the immune system works. At the same time that we are learning more about the unique characteristics of newborn and young infant immunity, the investigation of antibody immunodominance is rapidly expanding as we explore how the immune system develops preferential responses to some epitopes over others. The development of a vaccine that can provide broad protection against highly variable influenza A viruses will require thoughtful design that integrates findings from both of these fields of study. We propose our ability to meet this objective will benefit from the substantial overlap in approaches that have successfully induced protective immunity and promoted strong responses to conserved IAV epitopes. While there is still much to learn, the increasing information available offers encouragement that this goal can be reached.

## Figures and Tables

**Table 1:** Alterations in early life antibody responses relative to those seen in healthy adults.

**Initiation of the antibody response**	
Innate mediators of humoral immunity	↓ production of Th1-associated cytokines by DCs ↓ antigen presentation ↓ complement levels
B cell activation	↑ surface IgM expression ↑ initial proliferationAltered responses to TLR signaling despite similar levels of expression
GC localization	↓ CD62L and CCR7 on newborn B cells ↓ homing to lymphoid organs
**The germinal center response**	
B cells	↓ somatic hypermutation ↓ mutation and clonal diversity ↓ antigen presentation to obtain T cell help
T follicular helper cells	↓ differentiation and localization to the GC (likely due to ↓ transcription of CXCR5 and Bcl-6)↓ surface expression of CD40L ↑ representation of regulatory T cells
Follicular dendritic cells	Functional immaturity impairs establishment and maintenance of GC architecture
**Lasting antibody protection**	
Terminal differentiation	Preferential generation of MBCs ↓ adoption of plasma cell phenotype (↓ TACI expression)
Survival	↓ homing of LLPCs to bone marrow niche ↓ production of survival signals by BM stromal cells

## Data Availability

Not applicable.
